# Cost-Effectiveness of New Cardiac and Vascular Rehabilitation Strategies for Patients with Coronary Artery Disease

**DOI:** 10.1371/journal.pone.0003883

**Published:** 2008-12-09

**Authors:** Sandra Spronk, Johanna L. Bosch, Constance Ryjewski, Judith Rosenblum, Guido C. Kaandorp, John V. White, M. G. Myriam Hunink

**Affiliations:** 1 Department of Epidemiology, Erasmus MC, Rotterdam, the Netherlands; 2 Department of Radiology, Erasmus MC, Rotterdam, the Netherlands; 3 Department of Surgery, Advocate Lutheran General Hospital, Chicago, Illinois, United States of America; 4 Department of Health Policy and Management, Harvard School of Public Health, Boston, Massachusetts, United States of America; 5 Department of Surgery, University of Illinois College of Medicine, Chicago, Illinois, United States of America; MRC Biostatistics Unit, United Kingdom

## Abstract

**Objective:**

Peripheral arterial disease (PAD) often hinders the cardiac rehabilitation program. The aim of this study was evaluating the relative cost-effectiveness of new rehabilitation strategies which include the diagnosis and treatment of PAD in patients with coronary artery disease (CAD) undergoing cardiac rehabilitation.

**Data Sources:**

Best-available evidence was retrieved from literature and combined with primary data from 231 patients.

**Methods:**

We developed a Markov decision model to compare the following treatment strategies: 1. cardiac rehabilitation only; 2. ankle-brachial index (ABI) if cardiac rehabilitation fails followed by diagnostic work-up and revascularization for PAD if needed; 3. ABI prior to cardiac rehabilitation followed by diagnostic work-up and revascularization for PAD if needed. Quality-adjusted-life years (QALYs), life-time costs (US $), incremental cost-effectiveness ratios (ICER), and gain in net health benefits (NHB) in QALY equivalents were calculated. A threshold willingness-to-pay of $75 000 was used.

**Results:**

ABI if cardiac rehabilitation fails was the most favorable strategy with an ICER of $44 251 per QALY gained and an incremental NHB compared to cardiac rehabilitation only of 0.03 QALYs (95% CI: −0.17, 0.29) at a threshold willingness-to-pay of $75 000/QALY. After sensitivity analysis, a combined cardiac and vascular rehabilitation program increased the success rate and would dominate the other two strategies with total lifetime costs of $30 246 a quality-adjusted life expectancy of 3.84 years, and an incremental NHB of 0.06 QALYs (95%CI:−0.24, 0.46) compared to current practice. The results were robust for other different input parameters.

**Conclusion:**

ABI measurement if cardiac rehabilitation fails followed by a diagnostic work-up and revascularization for PAD if needed are potentially cost-effective compared to cardiac rehabilitation only.

## Introduction

Coronary artery disease (CAD) is the leading cause of mortality and morbidity in the United States[1]. Millions of Americans have a history of myocardial infarction or experience angina pectoris[1]. Many of these patients (on average 300 000 per year) enter a rehabilitation program and those who have undergone re-vascularization procedures undergo cardiac rehabilitation with the objective of improving exercise tolerance, symptoms, serum lipid levels, and psychosocial well-being, while reducing cardiac risk factors and mortality[2], [3]. Published guidelines for cardiac rehabilitation and secondary prevention programs advocate a multifaceted program that includes a monitored 12 weeks exercise training of 36 sessions (3 sessions per week) and the pursuit of modifiable risk factor reduction through education, counseling, reinforcement of medical therapies, behavior change and acceptance of personal responsibility on the part of the patient[4].

Patients with CAD, however, frequently have peripheral arterial disease (PAD)(range 19%–42%)[5], [6], of whom approximately 50% are symptomatic [5]. PAD hinders the cardiac rehabilitation program because patients are unable to achieve their target heart rate due to their limited walking distance. Almost half of the patients who start cardiac rehabilitation do not complete the program successfully[7], in large part due to the presence of PAD, and these patients are at increased risk for cardiac events during follow-up (20%–60% increased risk for MI)[8], [9]. Measurement of the ankle-brachial-index (ABI) at rest and post exercise is recommended as the initial screening test to make the diagnosis of PAD and using this to decide whether patients need a workup for PAD either if rehabilitation fails or prior to the rehabilitation program to improve the results of the program.

The aim of the present study was to evaluate the effectiveness, costs, and relative cost-effectiveness from the societal perspective of new rehabilitation strategies which include the diagnosis and treatment of PAD in patients with CAD undergoing cardiac rehabilitation.

## Methods

### Model structure

A Markov decision model was developed to compare current cardiac rehabilitation with new rehabilitation strategies for patients with CAD[10], [11]. Our primary cohort for analyses (the base-case) consisted of 64-year old male patients who entered a cardiac rehabilitation program.

**Figure 1:**
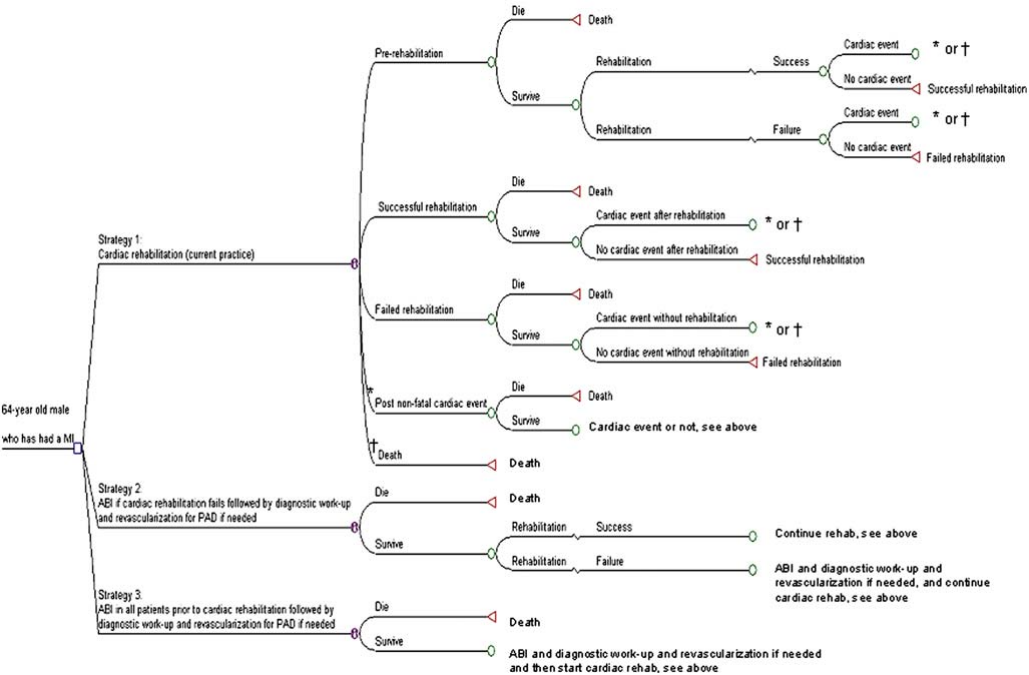
Schematic simplified representation of the Markov model. It shows three different rehabilitation strategies. Every strategy contains health states in which a patient can remain for more than one cycle. The health states are pre-rehabilitation (from which every patient starts), successful rehabilitation, failed rehabilitation, post non-fatal cardiac event, and death (i.e. cardiac death or non-cardiac death). All health states are only demonstrated in the upper strategy for simplification. MI = Myocardial infarction; PAD = peripheral arterial disease; CAD = coronary artery disease; ABI = ankle brachial index.

The strategies in the model were 1. Cardiac rehabilitation only; 2. Cardiac rehabilitation; if rehabilitation fails ABI measured at rest and post exercise and if needed a diagnostic work-up and revascularization for PAD, after which cardiac rehabilitation is continued; 3. ABI measured at rest and post exercise and if needed a diagnostic work-up and revascularization for PAD prior to cardiac rehabilitation, after which the rehabilitation is started. Figure 1 shows a schematic representation of the model. In the cardiac rehabilitation strategy, patients entered the program which they either completed successfully or they failed. If failure occurred due to PAD, no intervention took place and patients were followed in the outpatient clinic. Cardiac rehabilitation failure was defined as a discontinuation of the treadmill exercise program of 32 sessions or inability to reach target heart rate (individually determined during a stress test prior to cardiac rehabilitation). In follow-up, patients experienced a fatal or non-fatal cardiac event (i.e., defined as acute angina or non-fatal myocardial infarction) or they died from non-cardiac causes.

In the second strategy, patients also entered a cardiac rehabilitation program but now if patients failed, an ABI measurement at rest and post exercise was performed followed by diagnostic subtraction angiography or magnetic resonance angiography (proportion 1∶1) if PAD was present. Next, the lesion was treated with percutaneous intervention or bypass surgery depending on disease severity and level of disease. Suprainguinal percutaneous transluminal angioplasty (PTA) with stent placement, aorto-iliac grafting, infrainguinal PTA, and femoro-popliteal bypass were modelled as revascularization procedures. After revascularization, some patients had complications or procedural failures and were unable to continue cardiac rehabilitation, whereas most patients continued with their cardiac rehabilitation program.

In the third strategy, all patients underwent an ABI measurement at rest and post exercise prior to cardiac rehabilitation. If patients had symptomatic PAD, a diagnostic work-up and revascularization with PTA or bypass surgery was performed prior to cardiac rehabilitation.

For each of the three strategies, the model kept track of time and costs spent in one of the following health states: (a) pre-rehabilitation; (b) successful rehabilitation; (c) failed rehabilitation; (d) post non-fatal cardiac event; and (e) cardiac death or non-cardiac death. A Markov cycle tree was updated every 6 months after which patients' clinical status and costs were estimated to model life-time health benefits and costs.

### Data Sources

Effectiveness and cost data for the model were retrieved from the literature and from primary data collection. Table 1 and 2 show estimates from the best-available evidence of the included variables with probability distributions representing the uncertainty around the estimates and the data sources [5], [6], [12], [13], [14], [15], [16], [17], [18], [19], [20], [21], [22], [23], [24], [25], [26], [27], [28], [29], [30], [31], [32], [33], [34], [35], [36], [37], [38], [39], [40], [41], [42], [43], [44], [45]. Original patient data was collected in Lutheran General Hospital, Chicago, U.S., and included data from 231 consecutive men and women who started cardiac rehabilitation from January 2004–December 2004. Of the 231 patients, 125 patients (54%) completed cardiac rehabilitation successfully, 97 patients (42%) failed cardiac rehabilitation, and 9 patients (4%) were lost to follow-up. Of those 125/(125+97) = 56% who were not lost to follow-up completed rehab successfully, and the assumption is made that loss-to-follow-up status did not affect the chance of completing cardiac rehabilitation.

**Table 1:** Data included in the Markov model on rehabilitation strategies for patients with coronary artery disease.

*Variable*	*Base Case Value*	*Distribution*	*95% CI * §	*Literature or Database Source*
***Probabilities cardiac rehabilitation***				
Success current cardiac rehabilitation	0.56	Beta	0.13, 0.93	ALGH
Success cardiac rehabilitation after treatment PAD	0.71	Beta	0.22, 0.99	see text
Failure cardiac rehabilitation due to symptomatic PAD	0.18	Beta	0.04, 0.41	ALGH
PAD is cause after failure cardiac rehabilitation	0.40	Beta	0.09, 0.75	ALGH
Symptomatic PAD among cardiac rehabilitation patients	0.26	Beta	0.06, 0.54	ALGH, [5], [6]
***6-month rates of events during follow-up***				
Cardiac event after rehabilitation (fatal and non-fatal)	0.03	Beta	0.02, 0.04	[16], see text
Cardiac event without rehabilitation (fatal and non-fatal)	0.05	Beta	0.03, 0.05	[16], see text
Fatal cardiac event after rehabilitation	0.01	Beta	0, 0.05	[16], see text
Fatal cardiac event without rehabilitation	0.03	Beta	0, 0.07	[16], see text
***Probabilities PAD status***				
Suprainguinal disease conditional on the presence of PAD	0.56	Beta	0.01, 0.99	[17]
Suprainguinal lesion is suitable for angioplasty	0.51	Beta	0.36, 0.66	[15], [17]
Infrainguinal lesion is suitable for angioplasty	0.18	Beta	0.04, 0.41	[15], [17]
Lesion is suitable for surgery‡	0.95	Beta	0.82, 0.99	see text
Aorto-iliac lesion is occlusive vs. stenotic	0.20	Beta	0.01, 0.54	[18]
Femoro-popliteal lesion is occlusive vs. stenotic	0.36	Beta	0.01, 0.89	[14]
Vein is available for bypass surgery vs. PTFE is required	0.20	Beta	0.01, 0.53	ALGH
***Mortality rate for PAD procedures and imaging***				
Iliac PTA with selective stent placement	0.005	Beta	0, 0.01	[32], [38], [42]
Femoral or popliteal PTA	0.005	Beta	0, 0.01	[41], [45]
Aortic bifurcation grafts	0.02	Beta	0, 0.04	[40], [43]
Femoro-popliteal or infrapopliteal bypass	0.026	Beta	0, 0.05	[44]
Diagnostic imaging (angiography and magnetic resonance Imaging)	0.00033	Beta	0, 0.005	[22], [23]
***Probabilities systemic complications of PAD procedures*** *				
Iliac PTA with selective stent placement	0.007	Beta	0, 0.1	[42]
Femoral or popliteal PTA	0.003	Beta	0, 0.01	[39], [41]
Aortic bifurcation grafts	0.02	Beta	0, 0.04	[40]
Femoro-popliteal or infrapopliteal bypass	0.085	Beta	0.02, 0.18	[20]
***6-month patency in patients with PAD***				
Iliac PTA with selective stent placement †				
Stenosis	0.95	Beta	0.85, 0.99	[19]
Occlusion	0.80	Beta	0.58, 0.95	[19]
Femoro or popliteal PTA without stent placement				
Stenosis	1.0	Beta	0.95, 1.0	[24]
Occlusion	0.88	Beta	0.83, 0.95	[24]
Femoro or popliteal PTA with stent placement				
Stenosis	1.0	Beta	0.96, 1.0	[24]
Occlusion	0.99	Beta	0.95, 1.0	[24]
Aortic bifurcation grafts	0.98	Beta	0.96, 0.9	[25]
Femoro-popliteal or femoroinfrapopliteal bypass				
Autologous vein above-knee anastomosis	0.95	Beta	0.86, 0.99	[26], [27]
Autologous vein below-knee anastomosis	0.94	Beta	0.85, 0.99	[28]
PTFE, above-knee anastomosis	0.87	Beta	0.92, 0.96	[28], [29]
PTFE, below knee anastomosis	0.70	Beta	0.60, 0.79	[28], [29]

ALGH: Advocate Lutheran General Hospital; PTFE = Poly Tetra Fluor Ethylene, PTA = Percutaneous Transluminal Angioplasty, PAD = Peripheral Arterial Disease.

* Systemic complication is defined as all events that occurred within 30 days after the procedure and that required additional medical care.

† Patency estimates for iliac PTA with selective stent placement have been shown to equal those for iliac PTA with primary stent placement [29].

‡ In the Markov model, we assumed that 5% of the lesions were not suitable for surgery.

§ numbers are 95% CIs for the beta distributions.

**Table 2:** Health related quality of life and costs in U.S. Dollars.

*Variable*	*Base Case Value*	*Distribution*	*95% CIs/ranges * ††	*Literature or Database Source*
**Health-related quality of life weights**				
Pre-rehabilitation*	0.83	Uniform	0.51, 0.98	[12], [36]
After failed cardiac rehabilitation	0.83	Uniform	0.51, 0.98	See text
After successful cardiac rehabilitation	0.98	Uniform	0.80, 0.98	[12], [13], [30], [36]
After non-fatal cardiac event	0.83	Uniform	0.51, 0.98	[12], [36]
Systemic complications†	0.72	Uniform	0.48, 0.95	[37]
***Costs (US Dollars)*** §				
**Rehabilitation (6 months)**				
Scheduled visits cardiac rehabilitation	3 112	Lognormal		ALGH
Stress test**	95	Lognormal		ALGH
Follow-up visit**	75	Lognormal		ALGH
Transportation costs	117	Lognormal		ALGH
Patient time costs	481	Lognormal		ALGH, [47]
Total costs Cardiac rehabilitation if successfully completed	3 880	Lognormal	1385, 8636	ALGH
Total costs Cardiac rehabilitation if patient failed the program ¶	3 289	Lognormal	1201, 7249	ALGH
Post-Program after rehabilitation (per year)	1 257	Lognormal	446, 2800	[31]
***Diagnosis for PAD***				
Ankle-brachial index followed by treadmill walking	35	Lognormal	10, 90	ALGH
Diagnostic angiography/imaging∥	778	Lognormal	276, 1732	ALGH
***Revascularization for PAD***				
Aortic bifurcation grafts	32 942	Lognormal	11 711, 73 704	ALGH
Iliac PTA with selective stent placement ‡	9 618	Lognormal	4872, 17 193	[29], [32], [33]
Femoro-popliteal or infrapopliteal bypass	13 932	Lognormal	5019, 31 453	ALGH
Femoral or popliteal PTA	9 618	Lognormal	1243, 15 159	[33]
***Systemic complications after revascularization for PAD***				
Short-term costs	12 430	Lognormal	3004, 35 600	[33]
Annual long-term costs	13 715	Lognormal	3205, 37 411	[34]
Mortality from revascularization procedures	14 783	Lognormal	3571, 41 108	[33]
***Recurrent events***				
Non-fatal cardiac event first year	18 589	Lognormal	6537, 41 223	[31]
Non-fatal cardiac event annually thereafter	7500	Lognormal	1407, 21 904	[31]
Fatal cardiac event	20 971	Lognormal	7388, 4055	[31]

ALGH = Advocate Lutheran General Hospital; PTA = Percutaneous Transluminal Angioplasty; PAD = Peripheral Arterial Disease.

* Values based on responses on the EuroQol-questionaire [59], [60].

† Average Time Trade-off value among survivors of a myocardial infarction, used as a proxy for the effect on quality of life of a systemic complication[37].

‡ Assumes that in 43% of the cases a stent is placed [29].

§ Costs were converted to the year 2005.

¶ Based on the average number of sessions patients completed in ALGH.

∥ Costs are average costs of MRA and DSA because they were performed in the same proportion in ALHG.

** Costs are costs per event.

†† numbers are 95% CIs for the lognormal distributions and ranges for the uniform distributions.

### Effectiveness

In addition to estimates derived from the literature and the hospital database that were included directly in the model, some estimates were recalculated and several assumptions were needed. Hazard rates for fatal- and non-fatal cardiac events during follow-up for patients with and without cardiac rehabilitation, were calculated from probabilities derived from representative studies (i.e., those who compared cardiac rehabilitation to lifestyle changes only) which were included in a systematic review of Taylor and colleagues (Table 1)[16].

Long-term life expectancy was calculated on the basis of age-and sex-specific mortality rates from standard U.S. life-tables of the general population[35]. In addition, life-expectancy was adjusted for quality of life (i.e., Quality-Adjusted-Life Years (QALYs) using health-related quality-of-life weights (Table 2). To estimate the quality-of-life weight prior to rehabilitation and for successful completion of the program, we used the weighted average of health-related quality-of-life weights based on the literature[12], [36]. For patients who failed cardiac rehabilitation, quality-of-life weights were not available. We assumed that these patients had the same quality of life as prior to rehabilitation as they did not experience any benefit from the program (Table 2).

The proportion of patients who failed cardiac rehabilitation due to PAD was based on the ABI measured in a subset of our patient sample and based on data retrieved from the literature [5], [6]. All 231 patients were invited to the hospital for additional testing on a specific date, of which 39 patients responded. The patient characteristics between the subset and the non-responders were not statistically different (p>0.05). In the responders, the ABI was measured and PAD was defined as an ABI less than 0.90 [46]. The leg with the lowest ABI was used in the analyses.

In our patient sample, 7 out of 39 patients (18%) failed cardiac rehabilitation due to PAD, whereas of all patients who failed cardiac rehab (44%), there was 40% probability that PAD is the cause of cardiac rehab failure (i.e., 18%/44% = 40%). In strategies that included revascularization for PAD, we assumed that in the majority of patients (95%) revascularization was possible and that 90% of these patients would benefit from treatment. In our patient sample, 7 out of 39 patients (18%) failed cardiac rehabilitation due to PAD, therefore we modelled that 15% (i.e., 18% * 95% * 90%) underwent successful revascularization for PAD and subsequently continued their cardiac rehabilitation program. Thus, including the possibility of revascularization for PAD in the cardiac rehabilitation strategy, in total 71% (i.e., 56% plus 15%) of all patients completed the cardiac rehabilitation program successfully.

### Costs

Costs included in the model incorporated medical and non-medical costs and were assessed from the societal perspective (Table 2). Medical costs included costs of all diagnostic and therapeutic procedures, cardiac rehabilitation, costs for personnel, materials, equipment, associated hospital admissions during 6 months follow-up, and overhead. These costs were derived from the financial department of Lutheran General Hospital.

Non-medical costs included transportation costs and patient time costs. Transportation costs included parking costs and mean estimated gasoline costs. Patient time costs were determined by multiplying the hourly wage rate ($18.55/hour) by the number of hours or days spent in the hospital[47]. Time spent in the hospital was derived from our hospital database (e.g. cardiac rehabilitation 36 hours (60 min×36 sessions) and a bypass procedure was on average 6.5 days).

Costs of cardiac rehabilitation only included scheduled cardiac rehabilitation visits, a stress test, follow-up visits, transportation costs, and patient time costs.

To take into account time preference, future costs were discounted at the currently recommended nominal discount rate of 3% per year[48]. All costs were converted to the year 2005 U.S. dollars by using the medical care specific consumer price index obtained from the Bureau of labour Statistics[47].

### Analysis

Quality-adjusted-life expectancy, life time costs, and incremental cost-effectiveness ratios (i.e., additional costs divided by quality-adjusted-life-years gained) were calculated for all three strategies. Strategies were ordered according to increasing effectiveness (QALYs). A strategy was considered dominated if another strategy was both more effective and less costly. A strategy was considered extended dominated if another strategy achieved more effectiveness at a lower incremental cost-effectiveness ratio. After eliminating dominated and extended dominated strategies the incremental cost-effectiveness ratios (ICERs) were calculated as the difference in mean lifetime costs divided by the difference in mean QALYs for each strategy compared to the next best non-dominated strategy[49].

Furthermore, we transformed costs and QALYs into one comprehensive outcome measure: the net health benefit (NHB)[11], [50], [51]. The NHB was defined as lifetime effectiveness (QALYs) minus lifetime costs ($), the latter divided by the societal willingness-to-pay (WTP) threshold to save one QALY ($/QALY). The NHB is expressed in QALY-equivalents. Published estimates for WTP ranged from $20 000 to $100 000 per QALY gained. In our analysis we considered $75 000 per QALY gained as a commonly accepted threshold and varied the WTP between $50 000 and $100 000 in sensitivity analyses[51], [52]. For each of the two new strategies considered we calculated the gain in NHB compared to the NHB of cardiac rehabilitation only [53].

To explore the effect of uncertainty in our parameter estimates, we performed extensive one-way, two-way, and multi-way sensitivity analysis. Using probabilistic sensitivity analysis (second order Monte Carlo simulation), the uncertainty around the outcomes of the strategies was assessed [11], [54] by picking at random a value from each of the distributions of the parameter values, running the model with these values to get one set of outcome values, and repeating this 100 000 times[10].

Acceptability curves were used to express the uncertainty in the ICER from the probabilistic sensitivity analyses. These curves show for each predefined WTP-threshold the probability of cost-effectiveness for the three different strategies.

In value of information analysis we determined whether more information from future research is necessary to decrease the remaining uncertainty[55]. More research is not justified if the expected costs of further research exceed the expected benefit of that study. To estimate the total expected value of perfect information (EVPI) per patient, we calculated for each of the 100 000 Monte Carlo simulations from the probabilistic sensitivity analysis [53] the NHB of the optimal strategy per simulation, which is the expected NHB with perfect information. The EVPI is the difference between the mean expected NHB with perfect information from the probabilistic sensitivity analysis and the mean NHB with current information from the primary analysis. Next, we estimated the population EVPI, which is the total EVPI per patient multiplied by the expected lifetime of the technology (assumed to be 10 years) and multiplied by the annual number of future patients expected to benefit from more research (assumed to be 300 000, i.e. the annual number of patients who undergo cardiac rehabilitation in the U.S.) adjusted for the discount rate[53]. The EVPI expressed in NHB was reframed in terms of Net Monetary Benefit (NMB = NHB*WTP) which enables comparison with research costs. The EVPI expressed in NMB is the maximum amount worth spending on future research to decrease current uncertainty.

The model was developed in TreeAge (version TreeAge Pro suite 2007, TreeAge Software, Inc, Williamstown, Mass).

## Results

### Baseline analysis

Table 3 shows that an ABI measurement if cardiac rehabilitation fails followed by a diagnostic workup and revascularization for PAD if needed was the most favorable with an ICER of $44 251 per QALY gained. The NHB of this strategy was 3.38 (95% CI: 2.68, 3.95) at a WTP of $75 000 (Table 3). Intermediate outcomes presented in Table 4, showed that in a hypothetical cohort of 10 000 patients, the number of patients with a cardiac event during follow-up was lowest when an ABI measurement if cardiac rehabilitation fails was performed followed by a diagnostic work-up for PAD if needed. This benefit was partly diminished, however, by an increased number of patients with peri-procedural morbidity and mortality related to the PAD revascularization procedure.

**Table 3:** Cost, clinical effectiveness, and cost-effectiveness of (new) rehabilitation strategies for patients with coronary artery disease¶.

	Total Lifetime Costs* †	Quality-Adjusted Life Expectancy* (years)	Net Health Benefit (WTP = $75,000)	Incremental Costs per Quality-Adjusted Life Year ($/QALY)*
**Strategy 1:** Cardiac rehabilitation (Cardiac rehabilitation only)	29 724 (15 356, .57 271)	3.75 (2.97, 4.31)	3.35 (2.53, 3.98)	Reference
**Strategy 2:** ABI if cardiac rehabilitation fails followed by diagnostic work-up and revascularization for PAD if needed	32 658 (17 510, 60 818)	3.81 (3.19, 4.31)	3.38 (2.68, 3.95)	44 251
**Strategy 3:** ABI in all patients prior to cardiac rehabilitation followed by diagnostic work-up and revascularization for PAD if needed	41 032 (23 312, 71 289)	3.68 (2.98, 4.25)	3.13 (2.35, 3.78)	Dominated by strategy 1 and 2‡ §

QALY = Quality-Adjusted- Life Year; ABI = Ankle Brachial Index; PAD = Peripheral Arterial Disease; WTP = Willingness To Pay; Net health benefit = QALYs – (lifetime costs/ WTP).

* Future costs and life years were discounted at 3% per year.

† 2005 US dollars.

‡ More expensive and less effective than other strategy.

§ Compared to the next best strategy.

¶ Numbers are means (95% confidence intervals) derived from probabilistic sensitivity analysis.

**Table 4:** Intermediate Outcomes: number of fatal and non-fatal cardiac events* during follow-up and number of fatal and non-fatal peri-procedural complications in the base-case analysis in a hypothetical cohort of 10 000 patients.

Per 10 000 patients	Fatal cardiac event during follow-up	Non-fatal cardiac event during follow-up	Total cardiac event during follow-up	Fatal peri-procedural complications from PAD revascularization procedure	Non-fatal peri-procedural complications from PAD revascularization procedure	Total peri-procedural complications from PAD revascularization procedure
	*No. of patients*	*No. of patients*	*No. of patients*	*No. of patients*	*No. of patients*	*No. of patients*
**Strategy 1:** Cardiac rehabilitation	1838	1572	3410	0	0	0
**Strategy 2:** ABI if cardiac rehabilitation fails followed by diagnostic work-up and revascularization for PAD if needed	1742	1517	3259	42	46	88
**Strategy 3:** ABI in all patients prior to cardiac rehabilitation and diagnostic work-up and revascularization for PAD if needed	1826	1452	3278	64	91	155

Cardiac event = acute angina or non-fatal myocardial infarction; ABI = Ankle-Brachial index; PAD = Peripheral Arterial Disease.

* unrelated to PAD revascularization.

### Sensitivity analysis

In a two-way sensitivity analysis, we varied the success rate of the cardiac rehabilitation program by assuming that patients entered a combined cardiac and vascular rehabilitation program. In this analysis, we assumed that the success rate of current practice increased by 25%. This assumption was based on an 80% success rate as a result of the vascular component of the combined rehabilitation program in patients who would otherwise fail due to PAD. Thus, in this strategy an additional 14% (i.e., 80% of 18%) completed the program successfully. Therefore, in total 70% (i.e., 56% plus 14%) completed the new program successfully; hence, the increase in success rate of 25% (i.e., 70% versus 56%). This program would be comparable to the cardiac rehabilitation program, except for the aim of the program which is here both improving maximum walking distance and reaching THR by performing different appropriate exercise modalities. The same equipment will be used, but physical therapists need to be trained in order to know how to perform an individualized exercise prescription for aerobic and resistance training in patients with both CAD and PAD making the program more expensive ($400 additional costs). The results show, that this combined cardiac and vascular rehabilitation strategy would dominate the other two strategies with total lifetime costs of $30 246, a quality-adjusted life expectancy of 3.84 years and an incremental NHB of 0.06 (95%CI: −0.24, 0.41)compared to current practice.

In another two-way sensitivity analysis we changed both the “probability that PAD is the cause of cardiac rehabilitation failure” and the WTP value. If the probability would be lower, we expected that fewer patients would benefit from the strategy ABI if cardiac rehabilitation fails. Cardiac rehabilitation only was the preferred strategy below a threshold probability of 0.05 and a WTP value of $50 000 with a NHB of 3.35 QALYs. Doing a diagnostic work-up for PAD in all patients prior to the cardiac rehabilitation program would not be beneficial, which was mainly due to the higher costs of the diagnostic imaging and due to the peri-procedural complications. Multi-way sensitivity analysis demonstrated that for a WTP of $50 000 with a 50% increase in peri-procedural complications, 50% increase that the patient has PAD, and below a threshold of 0.10 that PAD is the cause of cardiac rehabilitation failure, cardiac rehabilitation only was the preferred strategy and performing a diagnostic work-up for PAD prior to cardiac rehabilitation in all patients was dominated by all other strategies.

For other parameters, we found that alternative assumptions either did not substantially affect the outcomes or affected all strategies similarly. If we lowered, for example, the original estimated rate of cardiac events after cardiac rehabilitation, the NHBs decreased for all strategies. Furthermore, varying the costs of fatal- and non-fatal cardiac events between 50% and 150% of the original estimates affected all strategies similarly and did not change the results of the NHBs.

### Probabilistic sensitivity analysis and Value of information analysis

Probabilistic sensitivity analysis demonstrated an incremental NHB of ABI if rehabilitation fails compared to cardiac rehabilitation only of 0.03 QALYs (95% CI: −0.17, 0.29), which implies considerable uncertainty around the outcome. Measuring the ABI in all patients prior to the rehabilitation program demonstrated a loss in NHB of −0.22 QALYs (95%CI: −0.49, −0.01) with 100% of the distribution below zero implying that this strategy is unlikely to ever be cost-effective compared to cardiac rehabilitation only.

**Figure 2:**
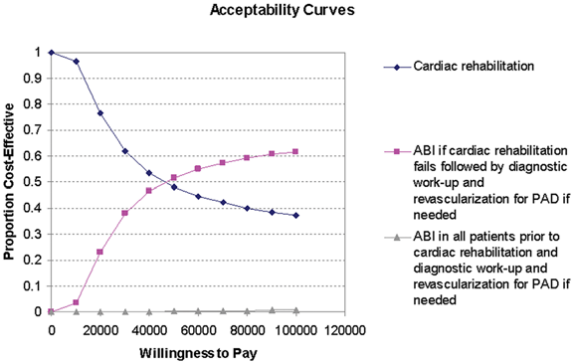
Acceptability curves for new cardiac and vascular rehabilitation strategies for patients with coronary artery disease. The x axis shows a range of values that society may be willing to pay for health benefits, and the elevation of the curve on the y axis denotes the probability that the strategy has an incremental cost-effectiveness ratio that is more favorable than the corresponding willingness to pay.

Figure 2 shows the acceptability curves for new cardiac and vascular rehabilitation strategies for patients with coronary artery disease. The probability that ABI if rehabilitation fails is cost-effective increases with an increasing threshold for the ICER. In the value of information analysis considering all three strategies the total EVPI per patient was $1 743 using a WTP of $75 000. This implies that an infinitely large future study is expected to increase the NMB per patient with $1 743. With the annual estimated number of patients that undergo cardiac rehabilitation of 300 000, an effective lifetime of a new rehabilitation strategy of 10 years, and a discount rate of 3%, the population EVPI was $2.4 billion.

## Discussion

In this study, we evaluated whether patients with CAD who currently enter a cardiac rehabilitation program would benefit more from the program if treatment for PAD is considered. The results suggest that a strategy with an ABI measurement if cardiac rehabilitation fails followed by a diagnostic work-up and revascularization for PAD if needed was the most attractive. A strategy that included an ABI measurement in all patients prior to the cardiac rehabilitation program did not increase QALYs compared to cardiac rehabilitation only. In a sensitivity analysis we assumed a combined cardiac and vascular rehabilitation approach in which we increased the success rate and the costs of current practice. This new program is expected to be more expensive but can also potentially prevent additional events in CAD patients during follow-up due to its higher success rate, which would lead to a gain in QALYs.

Current rehabilitation programs in the United States and in Western European countries consist of either cardiac rehabilitation for patients with CAD or vascular rehabilitation for patients with PAD. A combined program does not exist. Vascular programs range from hospital-based walking on a treadmill to home-based walking in the community until a mild or moderate level of pain is reached. These programs do not induce patients' target heart rate. We showed that it is attractive to develop a new rehabilitation program in which cardiac rehabilitation and vascular programs are combined, or revascularisation for PAD is considered, to decrease the failure rate of cardiac rehabilitation.

Due to continuously escalating medical costs, third-party payers demand evidence of cost-effectiveness and cost-related benefits of health care services and programs. With ABI measurement if cardiac rehabilitation fails followed by a diagnostic work-up and revascularization for PAD if needed, many secondary events can be avoided in patients with CAD. Nevertheless, we must interpret these results with caution because of the remaining uncertainty in our analysis. Future clinical research could reduce the uncertainty and patients could potentially benefit from more precise estimates of test characteristics, costs, and treatment effects. To assess whether the remaining uncertainty justifies future research, we performed a value of information analysis. The large population EVPI of $2.4 billion suggests that a substantial investment in future research would be justified.

One of the limitations of this study was that certain assumptions were needed in evaluating the rehabilitation strategies in a Markov model, which may have affected our results. For the assumptions, we specified a broad distribution for this model parameters and performed second order Monte Carlo simulation to select random values from this distribution in order to include this uncertainty. In addition, the available evidence regarding costs and effects was extrapolated over the entire remaining lifetime of patients. To explore how changes would affect the lifetime cost-effectiveness, extensive sensitivity analysis was performed and changing costs or effectiveness affected all strategies similarly. If we assumed, for example in our sensitivity analysis, an increase of the success rate of the cardiac rehabilitation probability by assuming a combined cardiovascular rehabilitation program, this strategy dominated the other two strategies. For many other assumptions, we demonstrated that varying the parameter values did not change the results substantially or changed the results for all strategies similarly while the conclusions remained the same. Another limitation of our study was the small subset of our patient sample who participated in the follow-up ABI measurement to determine the percentage failures due to PAD in our study group. However, the patient characteristics between the responders and non-responders were not significantly different and varying the percentage of failures due to PAD in a sensitivity analysis, we demonstrated that the results remained the same.

Cardiac rehabilitation programs remain underused in many countries. For example, in the U.S. only 10 to 20 percent of 2 million eligible patients per year who experienced a myocardial infarction or underwent cardiac revascularization procedures participated in a cardiac rehabilitation program[2]. Previous studies reported that factors such as poor patient motivation or co-existing illnesses were related to non-attendance of the cardiac rehabilitation program[56], [57], [58]. Many patients among non-participants could be physically inactive because of PAD, which could reduce patient's motivation to participate in a cardiac rehabilitation program and emphasizes the need to explore alternative strategies to diagnose and treat PAD in patients in cardiac rehabilitation programs.

In conclusion, the results suggest that a more aggressive approach to the diagnosis and treatment of PAD in CAD patients undergoing cardiac rehabilitation is warranted. ABI measurement in patients who fail cardiac rehabilitation followed by a diagnostic work-up for PAD and revascularization if needed, could potentially decrease secondary cardiac events and are likely to be cost-effective compared to cardiac rehabilitation only.
